# Supercurrent in van der Waals Josephson junction

**DOI:** 10.1038/ncomms10616

**Published:** 2016-02-02

**Authors:** Naoto Yabuki, Rai Moriya, Miho Arai, Yohta Sata, Sei Morikawa, Satoru Masubuchi, Tomoki Machida

**Affiliations:** 1 Institute of Industrial Science, University of Tokyo, 4-6-1 Komaba, Meguro, Tokyo 153-8505, Japan; 2 Institute for Nano Quantum Information Electronics, University of Tokyo, 4-6-1 Komaba, Meguro, Tokyo 153-8505, Japan

## Abstract

Supercurrent flow between two superconductors with different order parameters, a phenomenon known as the Josephson effect, can be achieved by inserting a non-superconducting material between two superconductors to decouple their wavefunctions. These Josephson junctions have been employed in fields ranging from digital to quantum electronics, yet their functionality is limited by the interface quality and use of non-superconducting material. Here we show that by exfoliating a layered dichalcogenide (NbSe_2_) superconductor, the van der Waals (vdW) contact between the cleaved surfaces can instead be used to construct a Josephson junction. This is made possible by recent advances in vdW heterostructure technology, with an atomically flat vdW interface free of oxidation and inter-diffusion achieved by eliminating all heat treatment during junction preparation. Here we demonstrate that this artificially created vdW interface provides sufficient decoupling of the wavefunctions of the two NbSe_2_ crystals, with the vdW Josephson junction exhibiting a high supercurrent transparency.

## Abstract (web summary)

Van der Waals heterostructures, made of stacked two-dimensional materials, hold promise for modern electronics. Here, the authors build van der Waals junction between superconducting two-dimensional materials and reveal that the junction works as Josephson junction.

## Graphical abstract

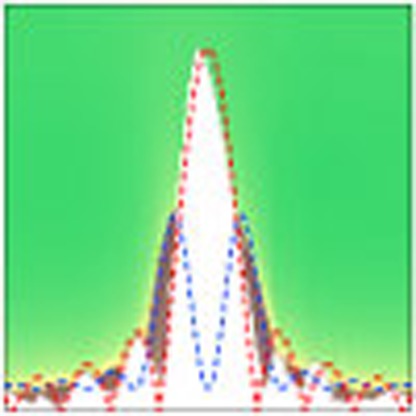

The micromechanical exfoliation of graphene from bulk graphite using Scotch tape, and its deposition on Si substrates^1^, has driven extensive research into the field of two-dimensional (2D) crystals, extending the field beyond graphene to include h-BN and transition metal dichalcogenides. In their bulk form, these exhibit a layered structure in which each individual layer is connected to its neighbours by van der Waals (vdW) forces, thus making it easy to create 2D crystals by cleaving at the vdW interface. The transfer of these 2D crystals onto other 2D crystals^2^, and their connection through vdW forces, makes it possible to create unique heterostructures—the so-called vdW heterostructures^3^. Indeed, many high-performance electronic devices based on a vdW heterostructure have already been demonstrated, including vertical field-effect transistors^4^,^5^, devices exhibiting strong light–matter interaction^6^,^7^ and ballistic Josephson junctions^8^. These outstanding properties are made possible by an absence of dangling bonds and lattice mismatch at the interface, which can also prevent interface defects and inter-diffusion. Furthermore, a vdW heterostructure provides an extra degree of freedom that is not possible to achieve in conventional heterostructures, namely, controlling the relative twist angle between the crystals at the vdW interface. Such twisting has been shown to alter the interlayer coupling at the vdW interface, leading to the discovery of a moiré superlattice in graphene/h-BN^9^,^10^,^11^, twist-controlled resonant tunnelling^12^ and decoupling in a twisted-bilayer system^13^,^14^.

The present study shows the decoupling of superconductivity at the vdW interface between two superconductors. We demonstrate that artificially created vdW contact between freshly cleaved layers of a NbSe_2_ superconductor can disrupt their wavefunctions and create a Josephson junction without the need for an additional non-superconducting layer. Fabricated vdW Josephson junction exhibits a high supercurrent transparency.

## Results

### Concept of a vdW Josephson junction

**Figure 1:**
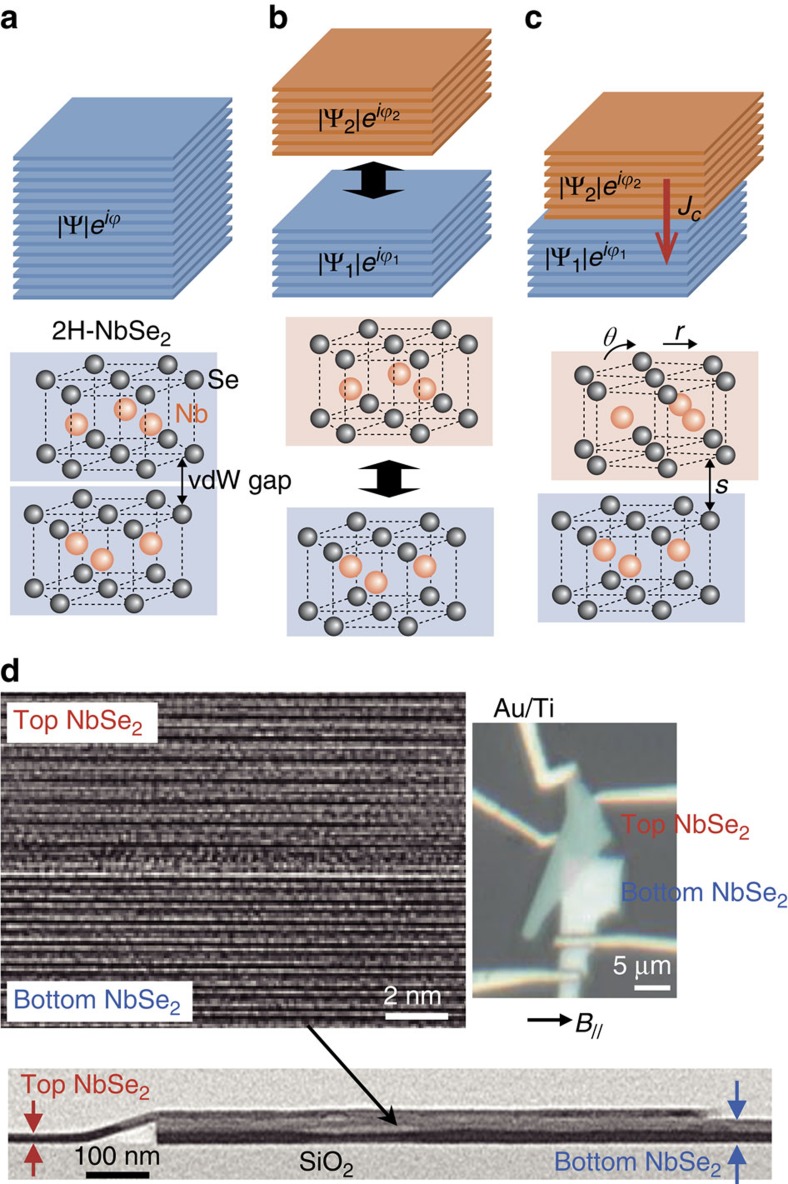
NbSe_2_/NbSe_2_ vdW Josephson junction. (**a**–**c**) Schematic illustration of the concept of a vdW Josephson junction. (**a**) Layered structure of bulk 2H-NbSe_2_ and the atomic arrangement of individual layers are depicted. In the superconducting state, the order parameter of the bulk crystal is described as |Ψ|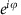, where |Ψ|^2^ denotes the density of Cooper pair and *ϕ* denotes the phase of the order parameter. (**b**) Exfoliation of the crystal into two different flakes at the vdW interface separates the system into two different order parameters, |Ψ_1_|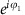 and |Ψ_2_|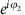. (**c**) Construction of a vdW junction between exfoliated NbSe_2_ crystals. The connection of two exfoliated crystals has three different degree of freedom; these are twist (*θ*), translation (*r*) and separation (*s*). If two different superconductors with different order parameters are connected, a supercurrent *J*_c_ will flow across the vdW junction. (**d**) Cross-sectional TEM images and an optical photograph of a vdW junction composed of top and bottom NbSe_2_ flakes 15 and 39 nm in thickness, respectively. Both high-magnification and low-magnification TEM images are presented. The low-magnification TEM image is compressed by 50% along the longitudinal direction. The Au/Ti contact (with 30 nm of Au and 50 nm of Ti) was fabricated for electrical measurements. The direction of the in-plane magnetic field *B*_//_ for the transport measurement is also indicated by an arrow.

The schematic illustration of a conceptual vdW Josephson junction is provided in Fig. 1a–c. Layered dichalcogenide 2H-NbSe_2_ was selected as the superconductor material, as it has particularly unique properties that make it ideally suited to a vdW Josephson junction. First, it has a layered crystal structure that allows individual monolayers of NbSe_2_ to be separated by at their vdW gap. Second, the superconducting transition temperature of NbSe_2_ varies significantly with interlayer modification such as a variation in stacking or interlayer separation^15^,^16^, suggesting that an atomic orbital overlap between layers plays a role in its superconductivity. When in a superconducting state as depicted in Fig. 1a, all of the electrons in NbSe_2_ are degenerated to a single bosonic state with an order parameter of |Ψ|*e*^*iϕ*^, where |Ψ|^2^ denotes the density of Cooper pairs and *ϕ* denotes the phase of the order parameter. Given that the layered structure of NbSe_2_ allows it to be exfoliated into thin flakes, each is characterized by different order parameters 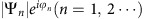 as depicted in Fig. 1b. Thus, when a vdW junction between flakes has the same atomic arrangement as the bulk, the interconnected layers work as one superconductor with a single-order parameter |Ψ|*e*^*iϕ*^. In other cases, such as when the vdW interface has a misalignment (*r*), misorientation (*θ*) and/or larger vdW gap (*s*) as depicted in Fig. 1c, the reduction in orbital overlap between layers reduces the interlayer coupling^13^,^14^. When this decoupling becomes sufficiently large, the superconducting state of the NbSe_2_ crystal created across the artificially connected vdW interface cannot be described by a single-order parameter, but rather requires different order parameters for each side of the vdW gap. This means that the flow of supercurrent across the interface follows the Josephson relation: *I*=*I*_c_sin(*ϕ*_1_−*ϕ*_2_), where *I*_c_ is the critical current.

### The vdW junction between NbSe_2_ flakes

The transmission electron microscopy (TEM) images in Fig. 1d show the layered crystal structure of a vdW junction created between atomically flat flakes, with thicknesses on the order of nanometres, exfoliated from a bulk crystal of NbSe_2_ by dry transfer (see the Methods section for further details of the fabrication method). The TEM cross-section image in Fig. 1d reveals that the layered structure of each flake has a period of ∼0.63 nm, which is very close to that of the original bulk 2H-NbSe_2_ crystal. Moreover, there is no significant change in the crystal structure of the NbSe_2_ layer, nor is there any evidence of oxide material at the vdW interface (Supplementary Note 1 and Supplementary Fig. 1). A nearly stoichiometric NbSe_2_ surface was also confirmed by chemical composition analysis (Supplementary Note 2 and Supplementary Fig. 2). This is in stark contrast to previous studies in which the NbSe_2_ surface was found to be Se-deficient after heat treatment^17^ or photo-oxidation^18^. The key to obtaining a high-quality vdW interface would therefore appear to be the elimination of all heat treatment procedures during device fabrication, as well as minimizing the extent to which the fresh NbSe_2_ surface is exposed to air and light. In other words, it is the carefully controlled environment used in this study that made it possible to eliminate surface oxidation and obtain an atomically smooth vdW junction. There is, however, a slight difference in the TEM contrast at the junction that is possibly caused by extra separation at the vdW junction as a result of the surface adsorption of molecules from the atmosphere onto the freshly cleaved NbSe_2_ surface. This is supported by the existence of adsorbed material at the vdW gap between NbSe_2_ flakes, as detected by energy dispersive X-ray spectroscopy measurement (Supplementary Note 3 and Supplementary Fig. 3).

### Josephson effect in the vdW junction

**Figure 2:**
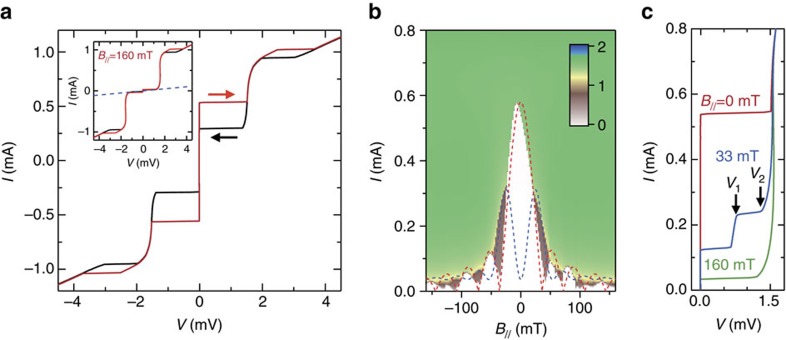
Electrical characteristics of a vdW Josephson junction. (**a**) Current–voltage (*I*–*V*) curve measured by sweeping the current at 2 K. Arrows indicate the sweep direction of the current. Inset: *I*–*V* curve measured with an in-plane magnetic field *B*_//_=160 mT. The dashed line indicates the quasi-particle tunnelling resistance within the sub-gap region. (**b**) Contour plot of *V* as a function of *I* and *B*_//_ at 2 K, showing a Fraunhofer pattern. The red and blue dashed lines present fitting results using *I*_c_(*X*) and 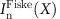. A colour bar shows the value of *V* in mV. (**c**) *I*–*V* curves measured at different *B*_//_ values. The arrows indicate current steps at the bias voltages *V*_1_ and *V*_2_ due to the Fiske resonance.

Figure 2a presents the current–voltage characteristics of the vdW junction under current biasing at 2 K; it is clear from the hysteresis of the *I*–*V* curve that its behaviour is typical of an underdamped Josephson junction. This means that the vdW interface introduces sufficient discontinuity in the superconducting order parameter for the Josephson effect to be observed. The data obtained from several other devices are presented in Supplementary Figs 4 and 5, which reveal the reproducibility of the Josephson effect in the fabricated vdW junction (Supplementary Note 4). Also, despite the use of such a simple fabrication method, a large critical current of *I*_c_=0.53 mA was observed. The junction area of 7.95 μm^2^ corresponds to a current density of 6,600 Acm^−2^, which is comparable to that of modern Josephson junctions used for single-flux quantum circuits^19^,^20^. Together with the hysteresis at the critical current of *I*_c_=0.53 mA, another voltage jump is observed at *I*=1.0 mA that is attributed to a breaking of superconductivity in the bulk NbSe_2_ rather than the junction itself (Supplementary Note 5 and Supplementary Fig. 6). Thus, using the slope of the *I*–*V* curve, the normal-state resistance is determined as *R*_N_=1.97 Ω (Supplementary Note 5 and Supplementary Fig. 6). The inset of Fig. 2a demonstrates how the application of a small in-plane magnetic field of *B*_//_=160 mT can suppress the *I*_c_; from this curve, the quasi-particle tunnelling resistance within the sub-gap region is determined as *R*_qp_=40 Ω. The ratio *R*_qp_/*R*_N_=20 therefore approaches the value for modern Nb-based Josephson junctions^20^. From the hysteresis of this curve, the re-trapping current *I*_r_ is found to be 0.3 mA. Using a *I*_r_/*I*_c_ ratio of ∼0.57, the junction's McCumber parameter *β*_c_ was found by comparing it with a microscopic model^21^ to be *β*_c_=0.6; the fact *β*_c_<1 defines the junction as an underdamped Josephson junction. By using the relationship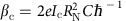, the junction capacitance *C* was calculated as *C*=1.2 μF cm^−2^, where *ħ* is Plank's constant and *e* is the elementary charge.

If the permeability is assumed to be that of a vacuum, the capacitance of the vdW junction corresponds to a thickness of 0.73 nm. Considering that a superconducting gap is opened in the Nb-derived band in NbSe_2_ (refs 22, 23) and that there is a finite extent of electron wavefunctions, the separation between the superconducting electron wavefunctions of the Nb plane in bulk NbSe_2_ should be smaller than its *c*-axis period of 0.63 nm. The larger value found at the vdW junction therefore provides further evidence that there is an extra separation present between the NbSe_2_ flakes here, and that this functions as an effective capacitor for the Josephson junction. It should also be noted that the second voltage jump at *I*=1.0 mA barely changes with the application of *B*_//_=160 mT, thus providing further evidence that this second jump is not related to the superconductor–insulator–superconductor junction itself, but rather to the breakdown of bulk superconductivity around the junction.

### Fraunhofer pattern

The application of a magnetic field in the direction of the junction plane induces a phase shift in the supercurrent, thereby inducing a variation in *I*_c_. The in-plane magnetic field dependence of the *I*–*V* curve at 2 K shown in Fig. 2b reveals a clear periodic modulation of *I*_c_ in relation to the magnetic field (that is, a Fraunhofer pattern). A second Fraunhofer pattern is also evident in the step-like increase of current observed in the *I*–*V* curve in Fig. 2c, wherein equally spaced steps at voltages *V*_1_ and *V*_2_ are clearly visible. These features are caused by Fiske resonance^24^, which is a coherently coupled mode between the a.c.-Josephson current and an electromagnetic wave within the junction. This combination of a Fraunhofer pattern and Fiske resonance provides yet another piece of evidence that a Josephson junction with a well-defined cavity was achieved. Both Fraunhofer patterns can be fitted with the analytical formula *I*_c_(*X*) and 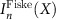
(see Methods section), where *X*=Φ*/*Φ_0_. Here Φ=(*d*+2*λ*)*LB*_//_ is the magnetic flux penetrating the junction along the magnetic field directions shown in Fig. 1d, *d* is the thickness of the non-superconducting region, *λ* is the London penetration depth of NbSe_2_ (5 nm)^25^, *L* is the width of the junction and Φ_0_ is the magnetic flux quanta. In this instance, it proved difficult to accurately determine the value of *d*; however, the TEM results provide a good indication that *λ* is much greater than *d*, and so Φ is assumed to be ∼2*λLB*_//_. This is validated by the fact that the calculated values of *I*_c_(*X*) and 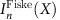 show good correlation with the experimental data in Fig. 2b, with the junction width determined from the fitting (*L*=5.5 μm) being very close to the actual junction width. From the fitting of the Fiske mode, the quality factor (*Q*) of the Josephson junction cavity was determined to be *Q*≈1,800; this value is quite large considering the fabrication method used to create the junction. These results further support the notion that the vdW interface functions as a Josephson junction with a well-defined junction area.

### Superconducting density of states

**Figure 3:**
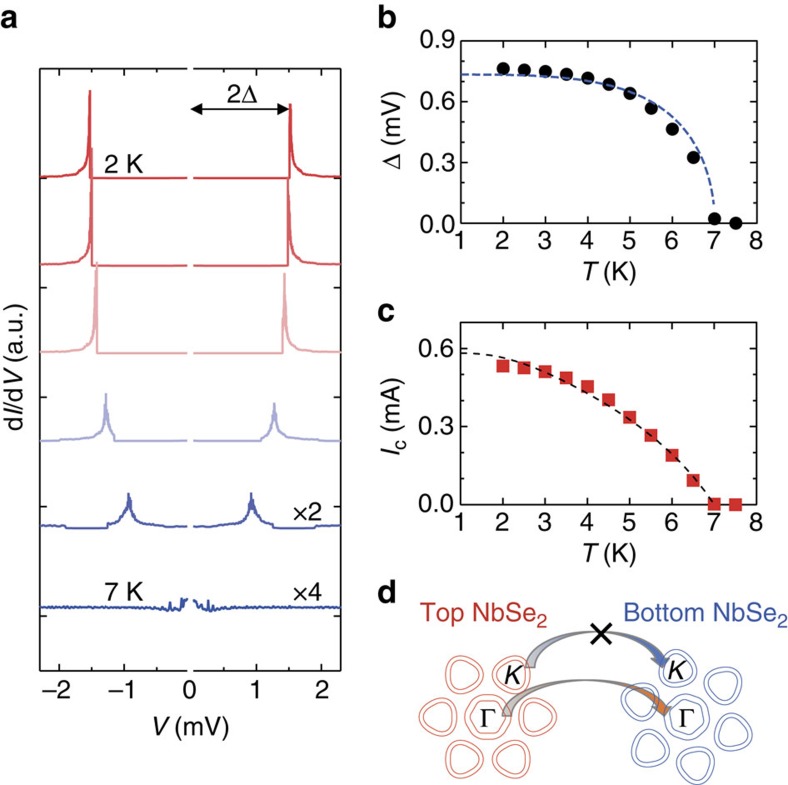
Relationship between temperature and superconductivity. (**a**) Variation in differential conductance d*I*/d*V* with respect to *V* measured at different temperatures. The length of the arrow equals two times the superconducting gap (that is, the arrow equals 2Δ) as determined from the position of the peaks in d*I*/d*V*. (**b**) Superconducting gap Δ versus temperature *T*, as determined from the data in **a**. The dashed line is calculated from BCS theory. (**c**) Critical current *I*_c_ versus *T*. The dashed line is calculated from AB theory. (**d**) Schematic illustration of the Fermi surfaces of the top and bottom NbSe_2_. Fermi surfaces of both the *K*-point and Γ-point are illustrated. The angle between the top and bottom NbSe_2_ is tilted 19°. The arrows indicate tunnelling between the top and bottom NbSe_2_.

To determine the superconducting density of states, *I*–*V* curves were measured at different temperatures, and the results obtained were numerically differentiated to obtain d*I*/d*V* as a function of bias voltage. As shown in Fig. 3a, the resulting d*I*/d*V* peaks are positioned at the value twice of superconducting gap, 2Δ. The single pair of peaks also suggests that there is only one superconducting gap contributing to the total conductance; the energy gap is plotted against temperature in Fig. 3b. From Bardeen–Cooper–Schrieffer (BCS) theory, the temperature dependence of the superconducting energy gap Δ(*T*) can be broadly expressed as 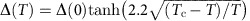, which fits well with the experimental data, as indicated by the dashed line. Here *T*_c_ is the critical temperature of the superconductivity. The experimental data can be further expanded with BCS theory to obtain Δ(0)=0.73 meV and *T*_c_=7 K; the latter shows good agreement with the value for bulk NbSe_2_ (∼7.2 K) and shows that there is no degradation of superconductivity at the vdW junction.

The temperature dependence of the critical current *I*_c_ of the symmetric Josephson junction was calculated from Ambegaokar–Baratoff (AB) theory^26^ using Δ(0) (see Methods section), and is plotted alongside the experimental data as the dashed line in Fig. 3c. This shows that the experimental data agree closely with AB theory and —assuming a symmetric single-gap superconducting electrode—gives a zero-temperature critical current of *I*_c_(0)=0.58 mA. These results are quite significant, as NbSe_2_ has up to now been generally regarded as a multi-gap superconductor^22^,^23^. In a *c*-axis-twisted Josephson junction, however, one has to take into account the anisotropy of the order parameter for coherent tunnelling^27^. Moreover, the superconducting gaps of NbSe_2_ exist in both the Γ and *ϰ* valleys of the *k*-space as illustrated in Fig. 3d, with the latter being more sensitive to any relative misorientation at the vdW interface. This ultimately means that the tunnelling of Cooper pairs at the *ϰ*-point is not preferential under twisting. From the optical micrograph shown in Fig. 1d, the misorientation angle between the two NbSe_2_ crystals was estimated as ∼19°. It would therefore seem likely that it is the occurrence of the Josephson effect at the more symmetric Γ-point that is responsible for the observed results.

## Discussion

From these comparisons, it has been revealed that a vdW interface created between exfoliated NbSe_2_ crystals functions as a Josephson junction, the properties of which are consistent with conventional BCS theory. From its *I*–*V* characteristics, the *R*_N_*I*_c_ product of the junction was determined as 1.04 mV at 2 K. This compares well with the theoretical maximum *R*_N_*I*_c_ value of *π*Δ/2=1.14 meV obtained through AB theory, indicating that the vdW interface is highly transparent to the phase coherent transport of supercurrent. Unlike most large-gap superconductors such as MgB_2_ and YBa_2_Cu_3_O_7_, which are difficult to integrate into a more complex heterostructure using evaporative techniques because of their low vapour pressure and structural complexity, this new approach of dry transferring cleaved layers provides an effective means of achieving single-crystalline superconductor heterostructures in which different materials are connected through a vdW junction.

## Methods

### Device fabrication

Nanometre-thick NbSe_2_ flakes were mechanically exfoliated from a bulk crystal (HQ Graphene Inc.) and deposited onto an 85-nm-thick SiO_2_ layer on a highly doped Si substrate. Using a dry-transfer technique^28^, a second NbSe_2_ layer was transferred onto the NbSe_2_ crystal; the cleaved surfaces of both NbSe_2_ crystals created a contact with vdW interaction in atmosphere. To minimize surface oxidation of both NbSe_2_ layers, the average time to fabricate this vdW junction was kept <1 h. A polymethyl methacrylate e-beam resist was subsequently spin coated onto the wafer and kept in a vacuum desiccator for at least an hour to remove any solvent. Next, using standard e-beam lithography and e-beam evaporation, a 30-nm Au/50-nm Ti electrode was fabricated. This entire device fabrication process was performed at room temperature without any intentional heating; this was crucial for minimizing surface oxidation of the NbSe_2_ crystals. The contact resistance of the Ti/NbSe_2_ interface created by this procedure was ∼150 Ω, a value which is three to four orders of magnitude smaller than that of a comparable device produced with heat treatment during the e-beam resist process.

### Ambegaokar–Baratoff theory

AB theory is used to calculate the temperature dependence of the critical current *I*_c_(*T*), and is expressed as^26^:


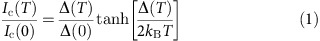


where *I*_c_(0) is the critical current at 0 K, Δ(0) is the superconducting gap at 0 K and *k*_B_ is the Boltzmann constant.

### Analysis of Fraunhofer pattern

The Fraunhofer pattern for the critical current of a Josephson junction is given as^26^:


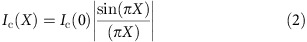


where *X*=Φ/Φ_0_ and Φ=(*d*+2*λ*)*LB*_//_ denote the magnetic flux, *d* is the thickness of the non-superconducting material, *λ* (5 nm) is the London penetration depth of NbSe_2_, *L* is the width of the junction and Φ_0_ is the magnetic flux quanta. The experimental data can be fitted by adjusting *I*_c_(0) and *L*.

The Fraunhofer pattern for Fiske resonance is expressed as^26^:


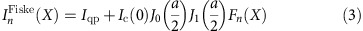



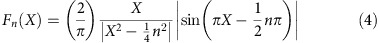


where *I*_qp_ is the quasi-particle current, *n* is the index of the Fiske mode and *J*_*n*_ a Bessel function of the first kind. The value *a* is obtained by solving the following equation:


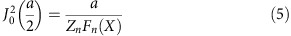



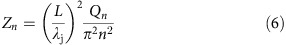


where *Q*_*n*_ represents the junction quality factor. By numerically calculating all these equations, the Fraunhofer pattern for the Fiske mode is obtained.

## Additional information

**How to cite this article:** Yabuki, N. *et al.* Supercurrent in van der Waals Josephson junction. *Nat. Commun.* 7:10616 doi: 10.1038/ncomms10616 (2016).

## Supplementary Material

Supplementary Information Supplementary Figures 1-6 and Supplementary Notes 1-5
