# Supplementary material for: Supercurrent in van der Waals Josephson junction
Source: Nat Commun. 2016 Feb 2;7:10616. doi: 10.1038/ncomms10616 (PMC4740878; doi:10.1038/ncomms10616)
Supplement: Supplementary Information — Supplementary Figures 1-6 and Supplementary Notes 1-5 [file ncomms10616-s1.pdf]

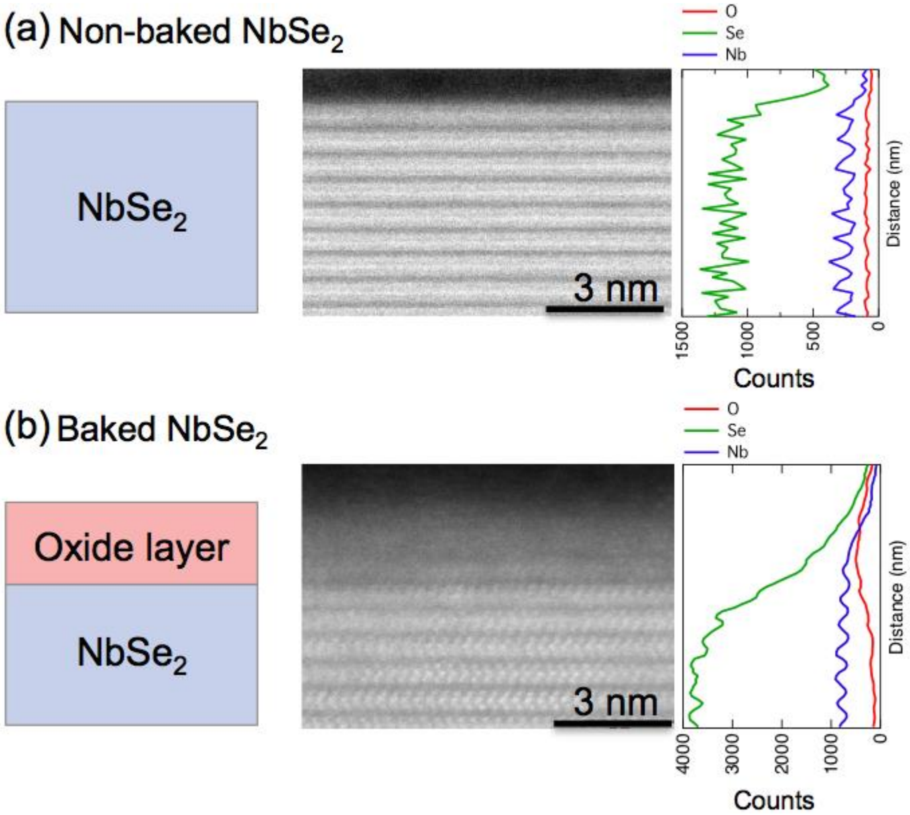

Supplementary Figure 1: TEM and EDX analysis of non-baked and baked $NbSe_2$ surfaces. Cross-sectional scanning transmission electron microscopy (STEM) image and energy dispersive X-ray spectroscopy (EDX) analysis of a (a) cleaved, non-baked $NbSe_2$ and a (b) cleaved, baked $NbSe_2$ surface. The schematic illustrations of the crystal structure are also depicted. The depth profile of the EDX signal for Oxygen (O), Selenium (Se), and Niobium (Nb) are plotted. Preparation conditions of both surfaces are described in Supplementary Note 1.

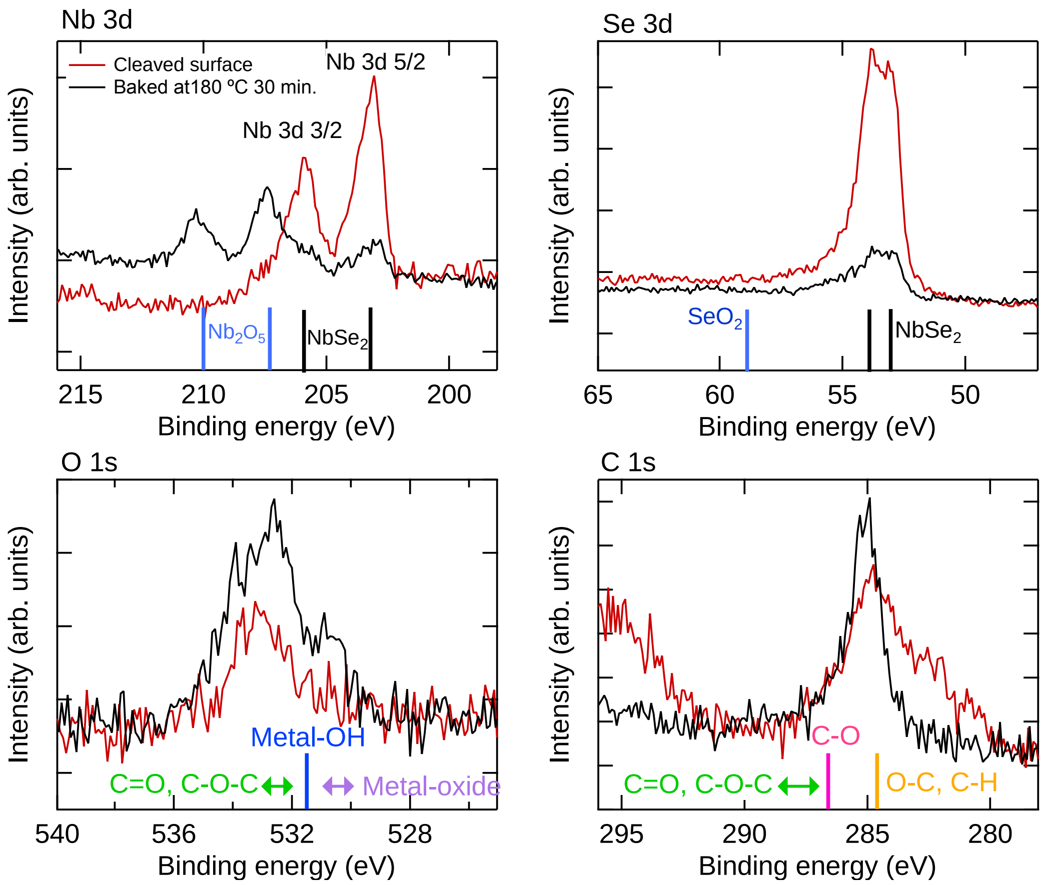

Supplementary Figure 2: XPS spectra obtained from non-baked and baked $NbSe_2$ surfaces.

X-ray photoelectron spectroscopy (XPS) spectra obtained from a cleaved, non-baked $NbSe_2$ surface (red line) and a cleaved, baked $NbSe_2$ surface (black line) are plotted. Spectra for Niobium (Nb) 3d, Selenium (Se) 3d, Oxygen (O) 1s, and Carbon (C) 1s are presented. The bars and arrows at the bottom of each figure indicate possible chemical species present on the surface.

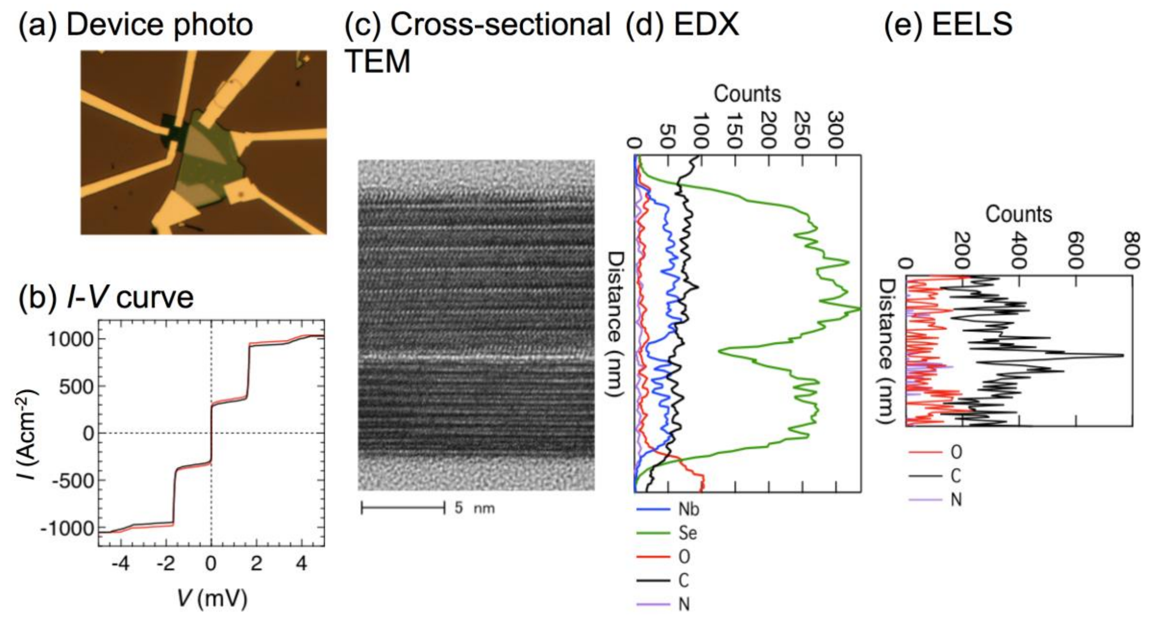

Supplementary Figure 3: Chemical composition analysis of the vdW junction. (a) Device photo of the $NbSe_2$/$NbSe_2$ van der Waals (vdW) junction used for the analysis. (b) Current-voltage (*I*-*V*) curve of the vdW junction measured at 2 K. The red and black lines indicate different current sweep directions. (c) Cross-sectional transmission electron microscopy (TEM) image of the vdW junction. (d) Energy dispersive X-ray spectroscopy (EDX) depth profile of the TEM image shown in (c). EDX signals for Niobium (Nb), Selenium (Se), Oxygen (O), Carbon (C), and Nitrogen (N) are plotted. (e) Electron energy loss spectroscopy (EELS) depth profile of the TEM image shown in (c). EELS signals for O, C, and N are plotted.

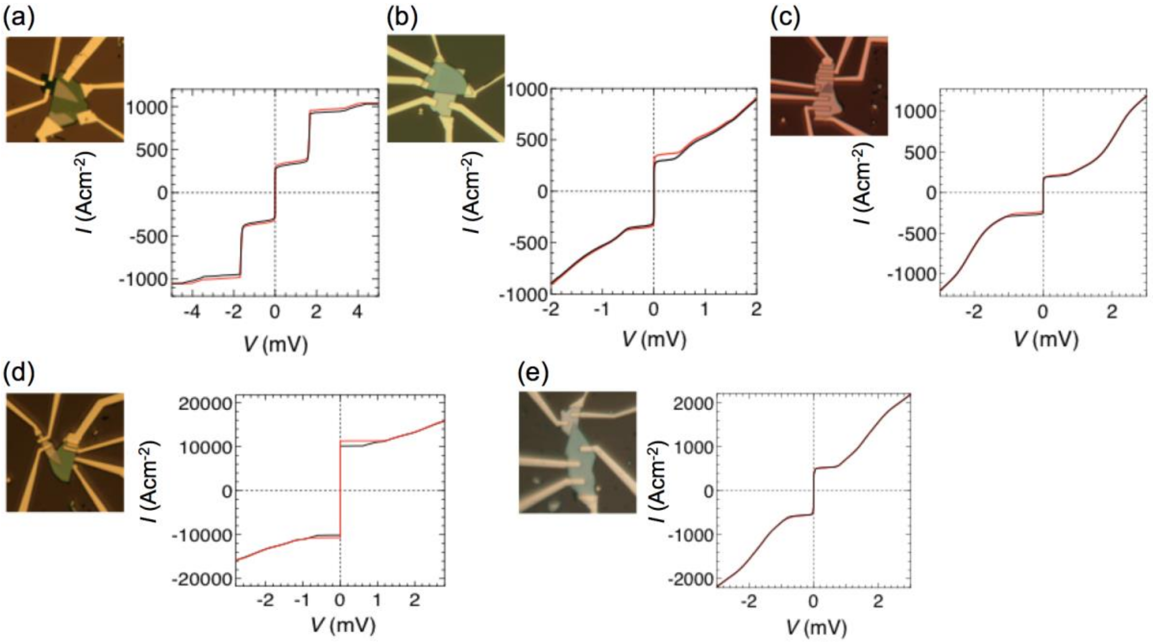

Supplementary Figure 4: *I-V* curves measured on low-resistance $NbSe_2/NbSe_2$ vdW junctions.

Current-voltage (*I-V*) curves measured on low-resistance $NbSe_2/NbSe_2$ van der Waals (vdW) junctions by sweeping the current at 2 K. The red and black curves indicate different sweep directions. The device photograph is presented together with the *I-V* curve.

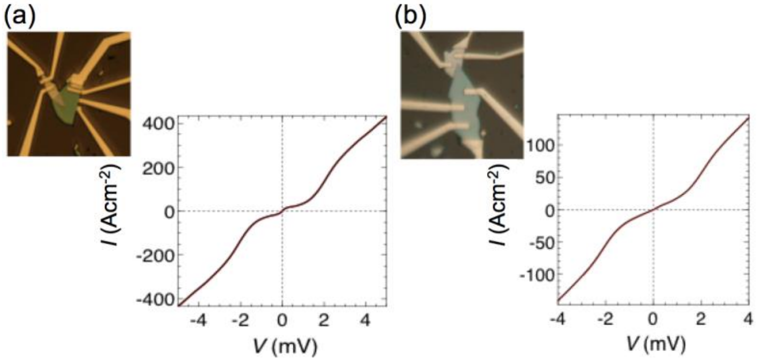

Supplementary Figure 5: *I-V* curves measured on high-resistance $NbSe_2/NbSe_2$ vdW junctions.

Current-voltage (*I-V*) curves measured on high-resistance $NbSe_2/NbSe_2$ van der Waals (vdW) junctions by sweeping the current at 2 K. The red and black curves indicate different sweep directions. The device photograph is presented together with the *I-V* curve.

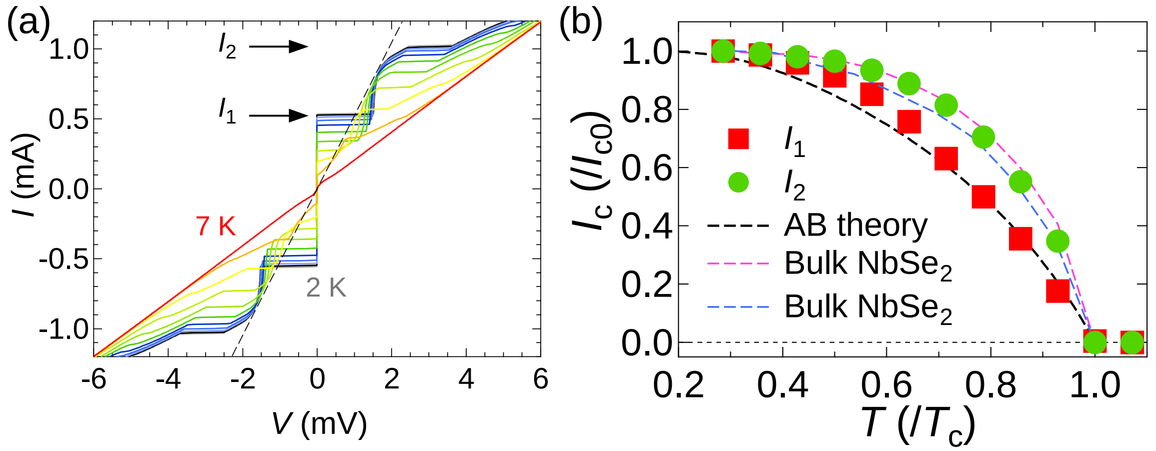

Supplementary Figure 6: Determination of the normal state resistance of a Josephson junction. (a) Change in the current-voltage (I-V) characteristics with temperature from 2 K to 7 K. The arrows indicate voltage steps at critical current values I_1 and I_2. Dashed line represents the normal-state resistance. (b) Dependence of the critical currents I_1 and I_2 on temperature. For comparison, the critical current calculated from Ambegaokar-Baratoff (AB) theory (black dashed line) and the temperature dependence of the critical current for breaking superconductivity of bulk $NbSe_2$ (pink and light blue dashed lines) are plotted together. Both critical current and temperature are normalized to their values at 2 K (I_{c0}) and at the transition temperature (T_c), respectively.

Supplementary Note 1: Oxidation of a cleaved $NbSe_2$ surface

The surface of $NbSe_2$ was evaluated through scanning transmission electron microscopy (STEM) and energy dispersive X-ray spectroscopy (EDX). Supplementary Figure 1 prresents the STEM images and EDX profile comparing a non-baked and baked $NbSe_2$ surface. The EDX profile for Oxygen (O), Selenium (Se), and Niobium (Nb) are plotted. Here, the non-baked surface is produced by the same device fabrication procedure used to create the Josephson junctions, but the surface is capped with carbon in place of another $NbSe_2$ crystal. In contrast, the baked surface is produced by spin coating the surface of $NbSe_2$ with a polymethyl methacrylate (PMMA) resist (without a capping layer), followed by baking at 180 °C for 30 min in a convection oven, and, thereafter, removing the PMMA resist using acetone. The EDX results clearly show a difference in the degree of surface oxidation between these samples, with a ~3-nm-thick surface oxide layer formed on the baked $NbSe_2$ sample. This oxide layer was confirmed through X-ray photoelectron spectroscopy (XPS) measurement to be Nb_2O_5. Oxidation of the non-baked $NbSe_2$ sample, on the other hand, is greatly reduced to below the resolution of EDX measurement.

Supplementary Note 2: XPS analysis of a cleaved $NbSe_2$ surface

The results of the X-ray photoelectron spectroscopy (XPS) analysis of the chemical composition of the cleaved $NbSe_2$ surface are shown in Supplementary Figure 2. Note that, because the cleaved $NbSe_2$ crystal was loaded into the XPS chamber less than an hour after exfoliation, its exposure time is considered comparable to that of the $NbSe_2$ surface used in the fabrication of the van der Waals (vdW) Josephson junction. From the Niobium (Nb) 3d, Selenium (Se) 3d, Oxygen (O) 1s, and Carbon (C) 1s spectra, it is clear that the most prominent peaks correspond to Nb and Se of $NbSe_2$; the integrated intensity confirms a Nb/Se ratio close to 1:2. No evidence was found of Nb_2O_5 or SeO_2 phases known to form on the surface of $NbSe_2$, with the only oxygen peaks observed being attributed to C=O or C-O-C bonding associated with the surface adsorption of hydrocarbon. Furthermore, no evidence was found of a signature metal-oxide peak that should be appear around 530~531 eV in the O 1s XPS spectra. However, after baking the same flake at 180 °C for 30 min with its surface covered with polymethyl methacrylate

(PMMA) resist, pronounced oxide peaks were observed. On the basis of these results, it is concluded that there is no significant oxidation of the $NbSe_2$ surface under the conditions used to fabricate the vdW Josephson junction.

Supplementary Note 3: Chemical composition analysis of the vdW junction.

Cross-sectional transmission electron microscopy (TEM) and energy dispersive X-ray spectroscopy (EDX) measurements were performed on different $NbSe_2$/$NbSe_2$ Josephson junction devices and the results are shown in Supplementary Figure 3. From the current-voltage (*I*-*V*) curve measured at 2 K, we confirmed that this device also shows the Josephson effect [Supplementary Figure 3(b)]. The cross-sectional TEM shows a layered structure of $NbSe_2$ crystals [Supplementary Figure 3(c)]. The EDX cross-section shown in Supplementary Figure 3(d) reveals that the Niobium (Nb) and Selenium (Se) signals decreases around the van der Waals (vdW) junction. Along with this, we do not see any Oxygen (O), Carbon (C), or Nitrogen (N) signals within the resolution limit of the EDX measurement. Comparing with EDX, electron energy loss spectroscopy (EELS) has superior sensitivity for lighter atoms. The EELS cross-sectional measurement shown in Supplementary Figure 3(e) reveals the presence of C at the vdW junction, and no signature of O. Thus it is suggested that there is some material in between the $NbSe_2$ flakes that separates the $NbSe_2$ flakes at the vdW junction. Comparing with the XPS data in Supplementary Figure 2, we attribute these C signals in the EELS measurement to molecules from the atmosphere that adsorbed onto the freshly cleaved $NbSe_2$ surface and remained in the vdW interface after vdW junction fabrication. The ion irradiation and high-energy electron beam irradiation during TEM specimen preparation and TEM measurement transform these molecules to hydrocarbons. The vdW junction between these naturally passivated $NbSe_2$ surfaces exhibits larger separation than its bulk crystal; this gives rise to decoupling of the wave functions of the two $NbSe_2$ flakes. The fact that no O-peak is detected at the vdW junction clearly suggests that there is no native metal-oxide (such as Nb-oxide and Nb-Se-O compound) at the junction. By carefully controlling the device fabrication process, the surface oxidation of $NbSe_2$ is suppressed.

Supplementary Note 4: *I-V* characteristics of extra devices.

More than 20 $NbSe_2/NbSe_2$ van der Waals (vdW) junction devices were fabricated to determine the nature of junction, and the data obtained from some of these devices are presented in Supplementary Figure 4(a,b,c) and 5(a,b). As can be seen from these figures, both the Josephson critical current and the hysteresis in the current-voltage (*I-V*) curve vary from device to device, even though the device fabrication conditions were the same. The Josephson effect is always present when the normal state junction resistance-area product *RA* is sufficiently low (i.e., less than ~300 $\Omega\mu m^2$); these devices are shown in Supplementary Figure 4(a,b,c). Conversely, with a high resistance junction such as that shown in Supplementary Figure 5(a,b), only a transport property of tunnel barrier was observed without zero bias Josephson current. Thus, it is crucial to reduce the junction resistance to observe the Josephson effect in a vdW junction. For fabricating such low resistance vdW junctions, the use of room-temperature device fabrication methods including exfoliation, dry-transfer, lithography, and metal evaporation play a key role.

Since there is no metal-oxide present at the vdW junction, we believe that the variation in resistance that was observed between the different devices is caused by a difference in the separation of the two $NbSe_2$ flakes at the vdW junction. This variation is therefore the limit of the present fabrication method, as it involves only the contact between two flakes of $NbSe_2$. It is likely that this separation depends on the quality of the fleshly cleaved $NbSe_2$ surface, the amount of surface adsorption on $NbSe_2$, and the force applied during transfer of the $NbSe_2$ flake. Although the junction resistance of the devices showed some variation despite using the same fabrication procedure, the Josephson effect is always present when the resultant vdW junction has a low normal-state resistance.

In addition, it was found that the junction resistance could be lowered by proper annealing, with the resultant low-resistance junction exhibiting the Josephson effect. For example, Supplementary Figure 5(b) was transformed to Supplementary Figure 4(e) after annealing at 400 K for 10 hours in a He atmosphere, while the device shown in Supplementary Figure 5(a) was transformed to Supplementary Figure 4(d) after annealing with a few mA of applied current for a few seconds. This demonstrates the robustness of the Josephson effect in a vdW junction.

Supplementary Note 5: Determination of normal-state resistance from the *I*-*V* curve of a Josephson junction.

Supplementary Figure 6(a) shows the change in the current-voltage (*I*-*V*) curve of the fabricated Josephson junction when the temperature was increased from 2 to 7 K. The dashed line represents the normal-state resistance, and as both *I*-*V* curves converge along this line, this was determined to have a value of R_N =1.97 Ω. Two different current jumps, I_1 and I_2, are shown in Supplementary Figure 6(b). It is evident from this that although the behavior of I_1 shows good agreement with Ambegaokar-Baratoff (AB) theory for a symmetric junction, as discussed in the main text, I_2 exhibits a significantly different temperature dependence. However, when the temperature dependence of I_2 is compared with the critical temperature required to break the superconductivity in bulk $NbSe_2$, the results are found to be in good agreement. It is therefore believed that I_1 is related to the junction, whereas I_2 is a contribution from the bulk $NbSe_2$. Note that bulk $NbSe_2$ does not usually experience hysteresis under the application of current, but as a long Josephson junction can produce an inhomogeneous current flow around it, some heating or phase slip can occur in the bulk material close to the junction area. Consequently, hysteresis can occur even in the bulk crystal.
